# Involvement of NY-ESO-1 and MAGE-A4 in the pathogenesis of desmoid tumors

**DOI:** 10.1097/MD.0000000000033908

**Published:** 2023-06-02

**Authors:** Kazuhiko Hashimoto, Shunji Nishimura, Yu Shinyashiki, Tomohiko Ito, Ryosuke Kakinoki, Masao Akagi

**Affiliations:** a Department of Orthopedic Surgery, Kushimoto Municipality Hospital, Wakayama, Japan; b Department of Orthopedic Surgery, Kindai University Hospital, Osaka-Sayama City, Japan.

**Keywords:** cancer testis antigen, desmoid tumor, melanoma-associated antigen A4, New York esophageal squamous cell carcinoma-1

## Abstract

The involvement of New York esophageal squamous cell carcinoma-1 (NY-ESO-1) and melanoma-associated antigen A4 (MAGE-A4) in soft-tissue sarcoma pathogenesis has recently been reported; however, their involvement in desmoid tumors (DTs) remains unknown. This study aimed to determine the involvement of NY-ESO-1 and MAGE-A4 in DTs. Immunostaining for β-catenin, NY-ESO-1, and MAGE-A4 was performed on DT biopsy specimens harvested at our institution. The positivity rate for each immune component was calculated. In addition, the correlations between the positivity rates for the immune molecules were investigated. The correlation between the positivity rate and age or longest diameter of each immune molecule was also investigated. β-catenin showed staining mainly in the tumor cell nuclei of DTs. Both NY-ESO-1 and MAGE-A4 showed staining in the nucleus, cytoplasm, and infiltrating lymphocytes of DT cells. The mean positive cell rates for β-catenin, NY-ESO-1, and MAGE-A4 were 43.9 ± 21.7, 30 ± 21.6, and 68.9 ± 20.8, respectively. A strong negative correlation was observed between β-catenin and MAGE-A4 positivity rates (*r* = −0.64). The positivity rates for NY-ESO-1 and MAGE-A4 showed a moderate positive correlation (*r* = −0.42). A very strong negative correlation was observed between age and the NY-ESO-1 positivity rate (*r* = −0.72). A weak negative correlation was observed between age and the MAGE-A4 positivity rate (*r* = −0.28). A medium negative correlation was observed between the longest tumor diameter and NY-ESO-1 positivity (*r* = −0.37). NY-ESO-1 and MAGE-A4 may be involved in the DT microenvironment. Thus, NY-ESO-1 and MAGE-A4 may be useful in the diagnosis of DT.

## 1. Introduction

Desmoid tumors (DTs), also known as invasive fibromas, are monoclonal neoplasms of myofibroblasts arising from muscle, tendon, and neural stromata.^[1]^ Morphologically, the tumor is composed of small, elongated, and spindle-shaped cells without characteristic cytoplasmic borders.^[2]^ According to the latest World Health Organization classification of soft tissue and bone tumors, a DT is an intermediate-grade neoplasm characterized by an invasive growth pattern into the surrounding normal structures and, very rarely, metastasis.^[3,4]^ The invasive nature of DTs is evidenced by the propensity of tumor cells to invade and engulf surrounding normal structures, which poses an imminent danger when they grow adjacent to major blood vessels and other vital organs.^[5,6]^ However, the pathogenesis of DTs remains unclear. Nonetheless, 85% of DTs harbor somatic mutations in the β-catenin gene *CTNNB1*.^[7]^ In addition, DTs have been shown to occur as part of inherited diseases, such as familial adenomatous polyposis syndrome.^[8,9]^ Both the *CTNNB1* and *APC* genes are part of the Wnt signaling pathway. Mutations in these genes upregulate β-catenin expression. Consequently, β-catenin accumulates in the nucleus and activates transcription factors in the Wnt pathway.^[10]^ Depending on the location of the lesion and the patient’s general condition, various treatment options exist, ranging from surgery to chemotherapy and radiation therapy.^[11]^ However, in many cases, the tumors recur more aggressively after treatment. Thus, the most desirable option for stable asymptomatic disease is careful monitoring of the patient, also known as the “wait-and-see policy.”^[12]^ Thus, DT treatment remains challenging, and its potentially invasive nature necessitates new treatment strategies.

Cancer and testicular antigen proteins (CTAs) are commonly expressed by testicular germ cells and cancer cells. A recent discovery that most of the 84 CTAs promote cancer cell growth and survival has attracted attention.^[13]^ CTAs are a group of tumor antigens whose normal expression is restricted to male germ cells in the testes and are not found in adult somatic tissues.^[14]^ In addition to their tissue-specific expression profile, common features of CTAs include their presence as a multigene family, frequent mapping to the X chromosome, induction of expression by hypomethylation and histone acetylation, immunogenicity in cancer patients, heterogeneous protein expression in various tumor types, and likely correlation with tumor progression.^[14]^ Notably, the CTA epitope is recognized by autologous T-lymphocytes that target cancer cells. Therefore, over the last decade, CTAs have emerged as therapeutic targets for the treatment of malignant diseases.^[15]^ New York esophageal squamous cell carcinoma-1 (NY-ESO-1) is an immunogenic CTA associated with innate and vaccine-induced immunity, which may lead to clinical cancer responses.^[16]^ Abnormal NY-ESO-1 expression has been reported in various neoplasms, including hepatocellular carcinoma, esophageal cancer, melanoma, and non-small-cell lung cancer.^[17]^ The melanoma antigen gene (MAGE) protein family is a large and highly conserved group of proteins with a common MAGE homology domain.^[1]^ MAGE-A is a CTA and Type I MAGE. In humans, Type I MAGE includes members of the MAGE-A, MAGE-B, and MAGE-C subfamilies clustered on the X chromosome.^[18]^ Similar to NY-ESO-1, the expression of many MAGE proteins is restricted to reproductive tissues; however, aberrant expression in various cancers has been reported. MAGE-A4 is widely expressed in many tumor types, including esophageal (60%), ovarian (47%), lung (19–35%), colorectal (22%), and breast (13%) cancers.^[18,19]^ However, the involvement of NY-ESO-1 and MAGE-A4 in the pathogenesis of DTs remains unclear. Therefore, this study aimed to characterize the significance of NY-ESO-1 and MAGE-A4 expression in DTs.

## 2. Material and methods

### 2.1. Immunohistochemistry

Immunostaining for β-catenin, NY-ESO-1, and MAGE-A4 was performed on DT biopsy specimens harvested at our institution. The study protocol was approved by the ethics committee of Kindai University (approval number, R03-021; approval date, April 27, 2021). The tissue sections were formalin-fixed and paraffin-embedded. Sections of 4 μm thickness were cut and mounted onto slides. Tissues were deparaffinized, rehydrated, and subjected to endogenous peroxidase inhibition using 3% hydrogen peroxide. Antigen activation was performed using antigen-specific heat treatment at pH 9 as follows: β-catenin, 95 °C for 64 minutes; NY-ESO-1, 100 °C for 64 minutes; and MAGE-A4, 95 °C for 36 minutes. Following heat activation, the tissue sections were incubated with the following primary antibodies: β-catenin antibody (rabbit polyclonal, ab16051; Abcam, Cambridge, UK), 37 °C for 32 minutes; NY-ESO-1 antibody (mouse monoclonal, E978; Santa Cruz Biotechnology, Santa Cruz, CA), 37 °C for 32 minutes; MAGE-A4 antibody (rabbit monoclonal, ab229011; Abcam), 37 °C for 16 minutes; and Ki67 antibody (mouse monoclonal, M7240; Agilent Technology, Santa Clara, CA), 37 °C for 16 minutes. The reaction was visualized using 3,3-diaminobenzidine (DAB Substrate Chromogen System; DAKO, Kyoto, Japan), and the sections were counterstained with hematoxylin. Testes were used as positive controls for NY-ESO-1 and MAGE-A4 expression. In all the immunohistochemical staining experiments, negative controls were prepared using IgG adapted for each stain to check for nonspecific binding. The slides were observed under a microscope (BIOREVO BZ-9000; KEYENCE, Osaka, Japan). Brown granules in the cytoplasm or nuclei indicated positive staining. Immune marker staining within the tumor was quantified in 4 representative high-power fields (40× magnification).^[20]^ The positivity rate for each immune component was calculated. The positivity rate was defined as the number of positive cells/total cell number and was quantified using the BIOREVO-BZ 9000 software (Keyence, Osaka City, Japan).

The correlations between the positivity rates for the immune molecules were also investigated. The correlation between the positivity rate and age or longest diameter of each immune molecule was also investigated. The study protocol was approved by the ethics committee of Kindai University (approval number, R03-021; approval date, April 27, 2021).

### 2.2. Statistical analyses

The positivity rate of each molecule was plotted and a correlation diagram was drawn. The coefficient of determination (*R*) was calculated by drawing an approximation line to examine the correlation between molecules. Pearson’s correlation method was used to examine correlations. The correlations between the clinical parameters and the positivity rate of each molecule were also investigated. Variables are presented as mean ± standard deviation. Correlations were defined as follows: very strong, 1.0≧|R|≧0.7; strong, 0.7≧|R|≧0.5; moderate, 0.5≧|R|≧0.4; medium, 0.4≧|R|≧0.3; weak, 0.3≧|R|≧0.2; and no correlation, 0.2≧|R|≧0.0. Analyses were performed using Stat Mate 5.05 (ATMS, Tokyo, Japan).

## 3. Results

### 3.1. Patient characteristics

The clinical characteristics of the study participants are summarized in Table 1. Four male and 5 female patients were enrolled in this study. The mean age was 37.0 (range, 11–84) years. The mean longest tumor diameter was 6.08 (range, 1.0–22.3) cm. Three and six tumors occurred in the extremities and trunk, respectively.

**Table 1:** Characteristics of the study population.

Factor	Patients, n
Age (yr)	
Median	37
Range	11–84
Sex	
Male	4
Female	5
Tumor site	
Arms	3
Trunk	6
Tumor size (cm)	
<5	5
5–10	3
>10	1
Treatment	
Wide resection	7
Marginal resection	2

### 3.2. Immunohistochemistry

**Figure 1.**
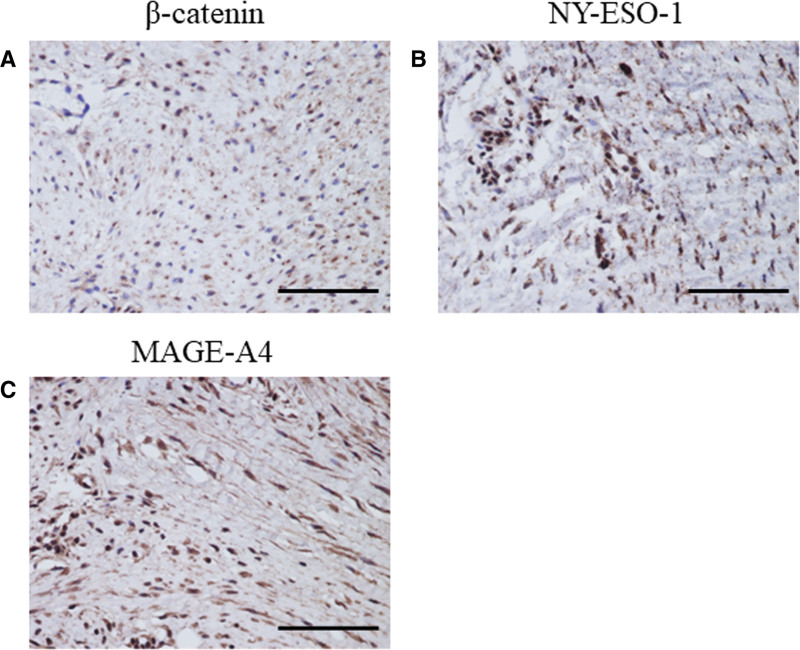
Immunostaining for β-catenin, NY-ESO, and MAGE-A4. Representative images showing β-catenin (A), NY-ESO-1 (B), and MAGE-A (C) staining. (A) β-catenin showed staining mainly in the tumor cell nuclei of DTs. (B and C) Both NY-ESO-1 and MAGE-A4 showed staining in the nucleus, cytoplasm, and infiltrating lymphocytes of DT cells. DT = desmoid tumor, MAGE-A4 = melanoma-associated antigen A4, NY-ESO-1 = New York esophageal squamous cell carcinoma-1.

β-catenin showed staining mainly in the tumor cell nuclei of DTs (Fig. 1A). Both NY-ESO-1 and MAGE-A4 showed staining in the nucleus, cytoplasm, and infiltrating lymphocytes of DT cells (Fig. 1B and C). The mean positive cell rates for β-catenin, NY-ESO-1, and MAGE-A4 were 43.9 ± 21.7, 30 ± 21.6, and 68.9 ± 20.8, respectively (Table 2).

**Table 2:** Immune molecule positive cell rates.

Immune molecule	β-catenin	NY-ESO-1	MAGE-A4
Positive cell rate (mean ± SD)	43.9 ± 21.7	30.0 ± 21.6	68.9 ± 20.8

MAGE-A4 = melanoma-associated antigen A4, NY-ESO-1 = New York esophageal squamous cell carcinoma 1, SD = standard deviation.

### 3.3. Correlation between immune molecules

**Figure 2.**
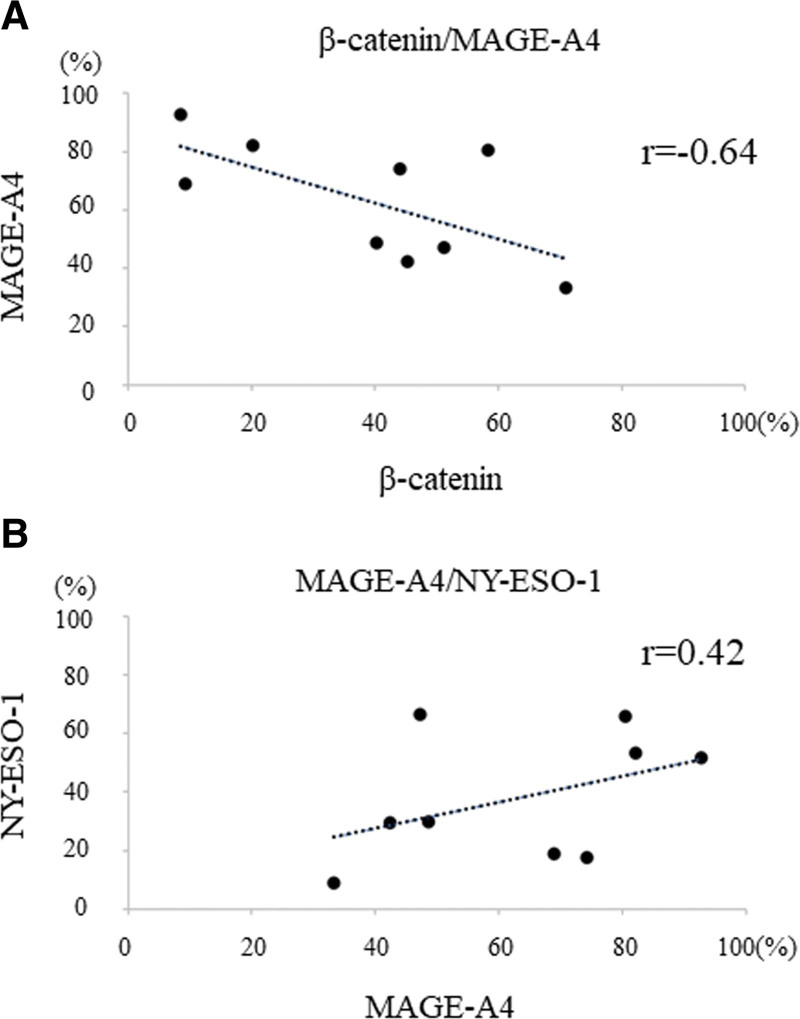
Correlation between positive cell rates of immunohistochemical markers. (A) A strong negative correlation was observed between β-catenin and MAGE-A4 positivity rates (*r* = −0.64). (B) NY-ESO-1 and MAGE-A4 positivity rates had a moderate positive correlation (*r* = −0.42). MAGE-A4 = melanoma-associated antigen A4, NY-ESO-1 = New York esophageal squamous cell carcinoma-1.

No correlation was found between β-catenin and NY-ESO-1 positivity rates (*r* = −0.09, data not shown). A strong negative correlation was observed between β-catenin and the MAGE-A4 positivity rate (*r* = −0.64, Fig. 2A). The positivity rates of NY-ESO-1 and MAGE-A4 showed a moderate positive correlation (*r* = −0.42, Fig. 2B).

### 3.4. Correlation between immune molecules and clinical features

**Figure 3.**
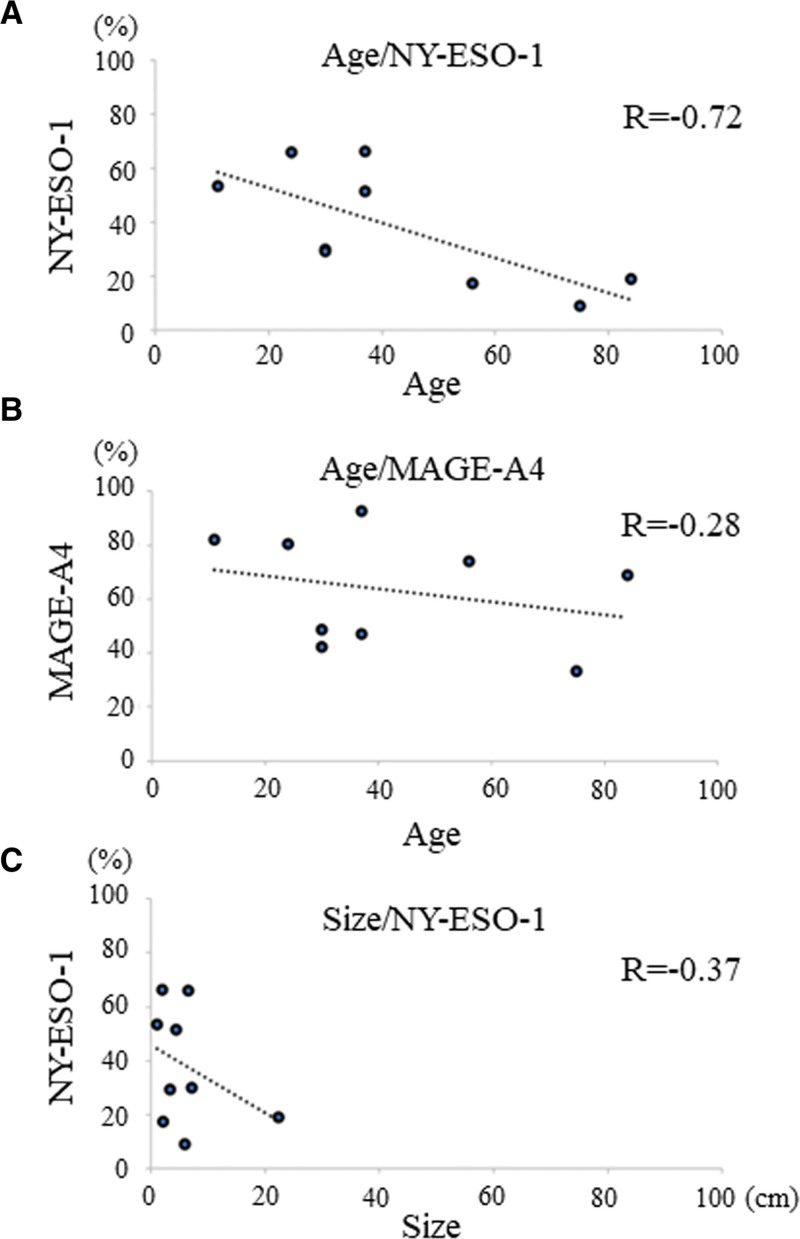
Correlation between immune molecules and clinical features. (A) Graphs showing the very strong negative correlation between age and the NY-ESO-1 positivity rate (*r* = −0.72) in desmoid tumors. (B) Graphs showing the weak negative correlation between age and the NAGE-A4 positivity rate (*r* = −0.28) in desmoid tumors. (C) Graphs showing the medium negative correlation between the longest diameter and NY-ESO-1 positivity rate (*r* = −0.37) in desmoid tumors. MAGE-A4 = melanoma-associated antigen A4, NY-ESO-1 = New York esophageal squamous cell carcinoma-1.

A very strong negative correlation was observed between age and the NY-ESO-1 positivity rate (*r* = −0.72, Fig. 3A). A weak negative correlation was observed between age and the MAGE-A4 positivity rate (*r* = −0.28, Fig. 3B). A medium negative correlation was observed between the longest tumor diameter and NY-ESO-1 positivity (*r* = −0.37, Fig. 3C). No correlation was observed between the longest tumor diameter and MAGE-A4 positivity (*r* = 0.0003; data not shown).

## 4. Discussion

The expression and significance of NY-ESO-1 and MAGE-A4 in DTs remain controversial. Therefore, we conducted this study to evaluate the expression and significance of NY-ESO-1 and MAGE-A4 in DT pathology.

Many trials examining immunotherapy have been conducted regardless of the sarcoma subtype, with promise in synovial sarcoma.^[21,22]^ Only 1 report showed that NY-ESO-1 was positive in 14.3% of DT cases.^[23]^ In the current study, all patients tested positive for both NY-ESO-1 and MAGE-A4, and the cell-positivity rates were relatively high (38% and 63%, respectively). A negative correlation was observed between the β-catenin and NY-ESO-1 cell positivity rates. Furthermore, a positive correlation was observed between the NY-ESO-1 and MAGE-A4 cell positivity rates. These findings suggest that the immune molecular mechanisms of NY-ESO-1 and MAGE-A4 are involved in the pathogenesis of DTs.

In recent years, significant advances in the characterization of the tumor microenvironment (TME) of soft-tissue sarcomas have described “hot tumors” with massive infiltration of immune cells and “cold tumors” with no significant immune infiltration.^[24]^ Petitprez et al^[24]^ established an immune-based classification system based on the composition of the TME and identified 5 distinct phenotypes: hypoimmune (A and B), hyperimmune (D and E), and hyper-vascular (C). Based on the results of the current study, DTs are immune-high tumors.

Older age and tumor size have been reported to be poor prognostic factors.^[25–27]^ Interestingly, previous studies have shown that, in high-grade sarcomas, patients who are NY-ESO-1 or MAGE-A4 positive have a significantly better overall survival than those who are negative for these 2 CTAs.^[3]^ The authors explained that the NY-ESO-1 and MAGE-A4 positive groups included many patients with synovial sarcoma and myxoid liposarcoma with relatively good prognoses because NY-ESO-1 expression induces T cell activity, leading to an antitumor response.^[3]^ The results of the present study were contrary to those of previous studies. This could be attributable to that NY-ESO-1 and MAGE-A4 may function differently in the TMEs of DTs and advanced soft-tissue sarcomas. It is also possible that the TME of naïve T cells encountering antigens in DTs can cause T cell dysfunction, such as functional memory, exhaustion, tolerance, anergy, and senescence.^[28,29]^

The present study has several limitations. First, the small sample size might have affected the significance of the findings and efficacy of the statistical results. Second, because only immunostaining was used in this study, NY-ESO-1 and MAGE-A4 gene expression could not be confirmed. Third, the direct effects of NY-ESO-1 and MAGE-A4 on the pathogenesis of β-catenin and DTs were not determined. Fourth, there are a few clinical considerations. However, we were able to investigate the correlations with immune molecules in terms of tumor size and age, and we believe that this study provides evidence that NY-ESO-1 and MAGE-A4 DT are involved in the pathogenesis of DTs.

## 5. Conclusion

The findings of this study suggest that NY-ESO and MAGE-A4 are involved in the pathogenesis of DTs; however, they may be exhausted in the TME. NY-ESO-1 and MAGE-A4 may be useful in the diagnosis of DT.

## Acknowledgments

We would like to thank Chikoto Tanaka for providing technical assistance.

## Author contributions

**Conceptualization:** Kazuhiko Hashimoto, Shunji Nishimura, Yu Shinyashiki, Tomohiko Ito, Masao Akagi.

**Data curation:** Kazuhiko Hashimoto, Shunji Nishimura, Tomohiko Ito, Ryosuke Kakinoki, Masao Akagi.

**Formal analysis:** Kazuhiko Hashimoto, Shunji Nishimura, Yu Shinyashiki, Tomohiko Ito, Ryosuke Kakinoki.

**Investigation:** Kazuhiko Hashimoto, Shunji Nishimura, Yu Shinyashiki, Tomohiko Ito, Masao Akagi.

**Methodology:** Kazuhiko Hashimoto, Yu Shinyashiki, Ryosuke Kakinoki, Masao Akagi.

**Project administration:** Kazuhiko Hashimoto, Yu Shinyashiki, Tomohiko Ito.

**Resources:** Kazuhiko Hashimoto, Shunji Nishimura, Tomohiko Ito, Ryosuke Kakinoki, Masao Akagi.

**Software:** Kazuhiko Hashimoto, Tomohiko Ito, Masao Akagi.

**Supervision:** Kazuhiko Hashimoto, Shunji Nishimura, Tomohiko Ito, Ryosuke Kakinoki, Masao Akagi.

**Validation:** Kazuhiko Hashimoto, Yu Shinyashiki, Tomohiko Ito, Ryosuke Kakinoki, Masao Akagi.

**Visualization:** Kazuhiko Hashimoto, Shunji Nishimura, Yu Shinyashiki, Tomohiko Ito, Masao Akagi,

**Writing – original draft:** Kazuhiko Hashimoto, Yu Shinyashiki, Tomohiko Ito, Masao Akagi.

**Writing – review & editing:** Kazuhiko Hashimoto, Shunji Nishimura, Tomohiko Ito, Ryosuke Kakinoki, Masao Akagi.
